# Acute Appearance of a Neck Mass in an 11-Year-Old Girl

**DOI:** 10.3390/pediatric12030022

**Published:** 2020-10-27

**Authors:** Ming-Hsiu Chiang, Yi-Ting Su, Liang-Ti Huang

**Affiliations:** 1 School of Medicine, College of Medicine, Taipei Medical University, Taipei 110, Taiwan; b101103050@tmu.edu.tw (M.-H.C.); b101103074@tmu.edu.tw (Y.-T.S.); 2 Department of Pediatrics, Wan Fang Hospital, Taipei Medical University, Taipei 110, Taiwan; 3 Department of Pediatrics, School of Medicine, College of Medicine, Taipei Medical University, Taipei 110, Taiwan

**Keywords:** infections, mumps, child, abscess, neck pain

## Abstract

Pustular abscess formation in the parotid gland is a rare complication following mumps virus infection. This is the second case report of pediatric parotid pustular abscess accompanied with mumps virus infection. Continuous antibiotics prescription without surgery is an eligible treatment for this patient.

## 1. Case Report

An 11-year-old girl, who had been relatively healthy, was admitted for a mass on her left neck. She had accidentally scratched herself behind the left ear a week before admission, and a mass had formed below the lesion 2 days after the injury. The patient visited a nearby clinic and received 3 days of cephalexin treatment for suspected bacterial infection. The mass decreased at first, but a relapse was observed after a short time; the mass became more swollen. She had no known past history and had not traveled abroad in the last 3 months. None of her family members nor classmates presented with similar symptoms. Her vaccination record revealed that she had followed the provisions by the Taiwan Center for Disease Control and was up to date. On review of her system, the patient appeared to have experienced dizziness, productive cough, and rhinorrhea for a few days.

**Figure 1:**
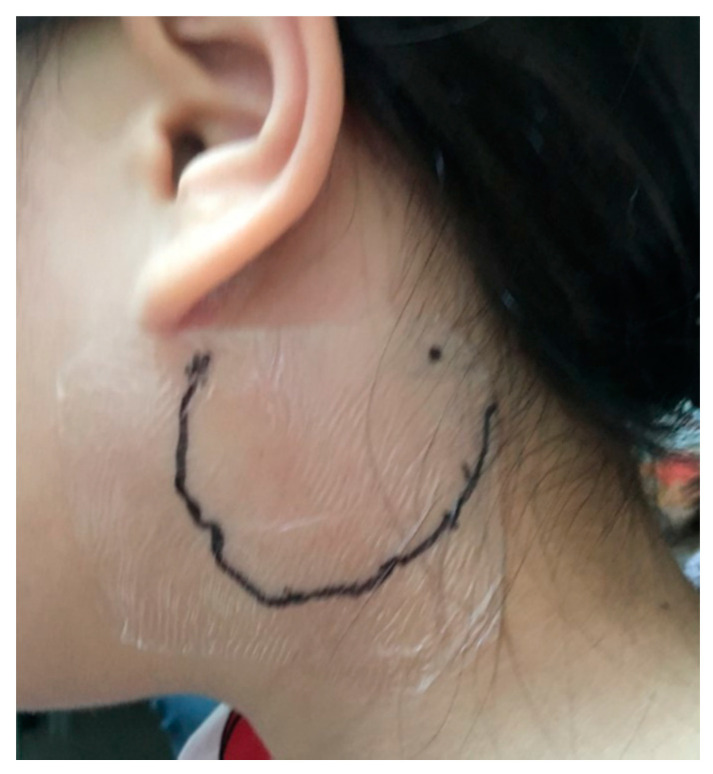
Patient photograph exhibiting mild reddish swelling on the left post-auricular area.

During physical examination, the patient complained that she could barely turn her neck because of the severe pain caused by the mass. Her body temperature was elevated to 38.5 °C after admission. The self-scratched wound produced no pustular discharge, and the scrape had healed well. The area of the mass on the left neck was 5 × 5 cm^2^. The mass was located at the left post-auricular area and appeared round, swollen, with mild redness (Figure 1). It was tender, movable, with a well-defined margin, and normothermic. Auscultation of her chest indicated bilateral coarse breathing sound with some rales and rhonchi.

A series of laboratory tests revealed no leukocytosis, neutrophilia, nor C-reactive protein elevation. Mid-stream urine test results and Epstein–Barr virus viral capsid antigen antibody serology results were normal. Chest radiography exhibited increased lung markings in bilateral lower lung fields. Oxacillin and gentamicin were prescribed empirically under the impression of pneumonia of unknown cause.

**Figure 2:**
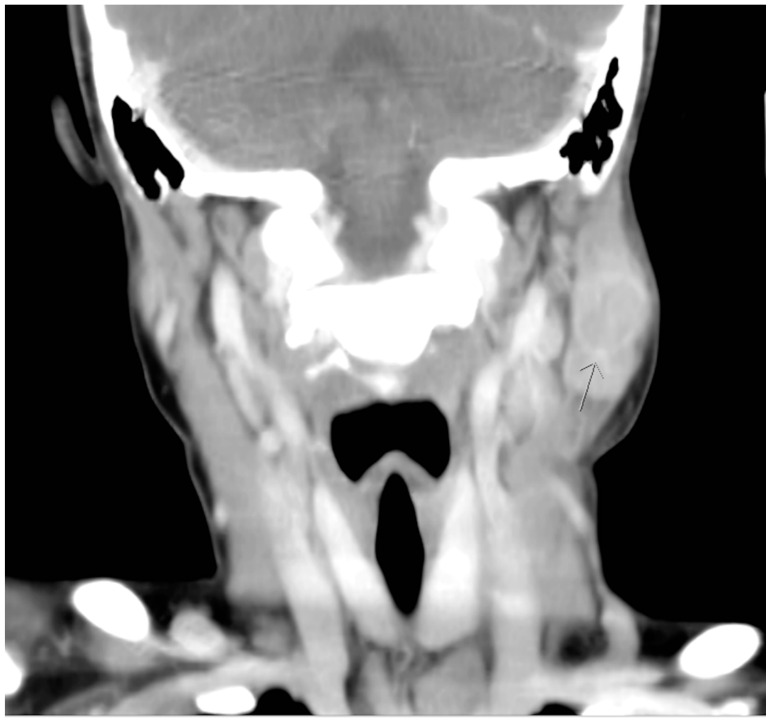
Coronal computed tomographic view revealing a hypodense cyst with ill-defined margin in the left parotid gland. (arrow head).

**Figure 3:**
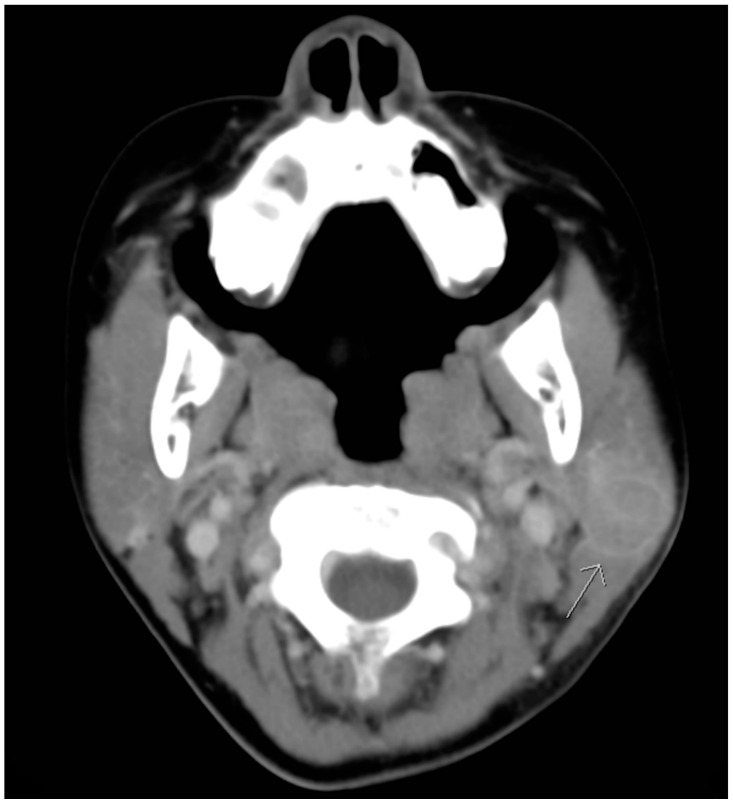
Transverse computed tomographic view revealing a hypodense cyst with ill-defined margin in the left parotid gland. (arrow head).

Although defervescence was observed on the seventh day after admission, the size of the mass was stationary. On the third day of hospitalization, azithromycin was prescribed based on the positive results of Mycoplasma pneumonia IgG and IgM serological tests. Nevertheless, the mass remained unchanged with prominent tenderness. Furthermore, a new round mass was observed on the ipsilateral part of the neck, which was located approximately 5 cm lower than the first mass; the size of this newly emerged mass was 2 × 2 cm^2^ and it was also hard, tender with well-defined margin. Computed tomographic (CT) scanning of the head and neck was therefore arranged, which revealed lymphadenopathy in the bilateral necks and a hypodense cyst with ill-defined margin in the left parotid gland. (Figure 2 and Figure 3). Based on the radiographic findings, a mumps virus immunoglobulin M (IgM) serology test was conducted.

Five to ten days after admission, both masses persisted with no signs of shrinkage even after the prescription of injected co-amoxiclav. We performed sono-guided aspiration following consultation of the Division of Pediatric Infectious Diseases and Division of Pediatric Surgery. Hypoechogenic fluids were evidenced under the bigger neck mass. A small fluid sample of the abscess was aspirated, and the cytology indicated numerous neutrophils, histiocytes, and lymphocytes. The results from aspiration culture were all no growth. The mumps IgM serology test was positive on the 11th day after admission, and mumps infection was thus diagnosed. Under co-amoxiclav treatment for 1 week, the size of the bigger neck mass decreased to 1.5 × 2.5 cm^2^, and the smaller mass disappeared. Meanwhile, no respiratory symptoms were observed. In view of her improved clinical condition, the patient was discharged from the hospital. No mass was detected after 1 week of follow-up in the outpatient department.

This case report has been approved by expedited review process of the TMU-Joint Institutional Review Board and the registered number is N202003023.

## 2. Discussion

Differential diagnosis of neck mass in paediatrics is broad and it could be classified into three categories, which are congenital, inflammatory, and neoplastic. As the patient reported some respiratory symptoms at admission, our first impression for the patient’s unilateral neck mass was acute lymphadenopathy, as a response to her respiratory infection. Owing to her comprehensive immunization records, it was not until a newly emerged neck mass and persistent fever manifestation that we started considering the possibility of mumps infection. Using serologic tests, which measure the concentrations of IgM antibody, the patient was diagnosed as having mumps infection.

Both sonography and CT scanning revealed abscess formation within the neck mass, which is unusual for mumps infection. Abscess formation within the parotid gland is known as suppurative parotitis and is mostly caused by *Staphylococcus aureus* [1]. In addition to hematogenous bacterial seeding, it can also occur if infections ascend from the oral cavity. As a result, decrease in salivary flow and poor oral hygiene are potential risk factors. Parotid abscess can be fatal as the infection may break through the parotid fascia and spread to the neck and head area, causing deep neck space infection [2]. In addition, parotid abscess can spread as necrotizing fasciitis and be life-threatening [3]. Ultrasound is the first-line diagnostic tool, and antibiotics with adequate hydration are the mainstay of treatment [4]. To the best of our knowledge, this is the second case report of a patient having mumps infection with concomitant parotid abscess [5]. We have suspected that the abscess formation could be related with the self-scratched wound. Although the culture result from the sono-guided aspiration was negative, for which we conjectured that it could be attributed to previous antibiotics usage, we still prescribed antibiotic treatment in case of intraparotideal or glandular parenchyma bacterial infections. After the discovery of the parotid abscess, we consulted a pediatric surgeon on the necessity of surgical intervention, and the doctor suggested continuing antibiotic treatment considering the patient’s stable vital signs. Unlike in the first case report of this phenomenon, we did not perform operation on the patient. Instead, we performed sono-guided aspiration in order to identify the infectious source and administered co-amoxiclav injection for 1 week, and the mass gradually regressed.

The mumps virus is a contagious childhood disease predominantly affecting children between the ages of six and seven [6]. Although routine vaccination against mumps has markedly reduced its incidence, sporadic outbreaks still occur in vaccinated populations worldwide because of decreased immunity, waning antibody levels, and change in the circulating virus genotypes [7]. A population-based study conducted in Taiwan revealed that the overall seroprevalence of mumps was 71%, with adults having higher seropositivity than children. A decline of seropositivity was observed after the injection of a second universal MMR vaccine before entry to elementary school, and it reached its lowest point in the age group of adolescents between 17 and 18 years old [8]. This finding was compatible with the observation of previous large mumps outbreaks in a highly vaccinated student population [9].

There are some limitations in this case report. First, the infectious source for this girl was ambiguous since there were no other cases reported in her family members or close contact persons. One possible explanation may be an accidental infection through the self-scratched wound two days before the appearance of the mass. Therefore, the possibility of the parotid abscess caused by bacteria could not be ruled out. The second limitation is that we did not perform real-time PCR in diagnosing the mumps infection. However, according to a 2009 cohort study, the positive predictive value of IgM serology test was 97.4% with only one false-positive case among 38 mumps real-time PCR-confirmed cases [10]. Moreover, the clinical symptoms of the patient were compatible with mumps infection, making the diagnosis of mumps infection plausible.

In conclusion, we presented a case of mumps virus infection with concomitant parotid abscess and Mycoplasma pneumonia. Adequate antibiotics usage with appropriate imaging studies are essential for treating and diagnosing parotitis that may accompany parotid abscess.

## Abbreviations

CT	computed tomographic
IgM	immunoglobulin M
